# Vulnerable children, stigmatised smokers: The social construction of target audiences in media debates on policies regulating smoking in vehicles

**DOI:** 10.1177/1363459316633279

**Published:** 2016-07-24

**Authors:** Josh Bain, Heide Weishaar, Sean Semple, Sheila Duffy, Shona Hilton

**Affiliations:** University of Glasgow, UK; The University of Aberdeen, UK; Action on Smoking & Health (Scotland), UK; University of Glasgow, UK

**Keywords:** children, media analysis, second-hand smoke exposure, smoking in vehicles, stigmatisation

## Abstract

Following restrictions on smoking in vehicles carrying children in several countries, legislation to safeguard minors from second-hand smoke exposure in vehicles is under-consideration or has been implemented across the United Kingdom. This article presents the first investigation into social constructions of children, smokers and smoking parents in newsprint media and coverage of debates about protecting children from exposure to second-hand smoke in vehicles. Using Scotland as an example, articles on children’s exposure to second-hand smoke published between 1 January 2004 and 16 February 2014 in three Scottish newspapers were identified using Nexis UK. In all, 131 articles were thematically coded and analysed. Children were portrayed as vulnerable and requiring protection, with few articles highlighting children’s ability to voice concerns about the dangers of smoking. Smokers and smoking parents were mainly portrayed in a factual manner, but also frequently as irresponsible and, in some cases, intentionally imposing harm. Individual smokers were blamed for their recklessness, with only a small number of articles mentioning the need to assist smokers in quitting. Supporters of legislation focused on corresponding discourse, whereas critics directed debates towards established arguments against policy, including individual freedom, privacy and problems of enforcement. Focusing on children’s vulnerability to second-hand smoke might have increased support for legislation but risked a side effect of smokers being stigmatised. The media and supporters of public health policy are encouraged to consider appropriate approaches to raise awareness of the health harms of second-hand smoke to children while avoiding unintended stigmatisation of those in which they want to encourage behaviour change.

## Introduction

Research has shown that the way social groups are constructed in the media can influence public opinion, political agenda setting and the development of public policies (Benelli, 2003; Brodie et al., 2003; McAuliffe Straus, 2004; Schneider and Ingram, 1993). Scholars hypothesise that public understanding of, and acceptance of policy responses to, health issues is considerably shaped by the news media (Gorini et al., 2011; Malone et al., 2000) and that policymakers are influenced by the media’s social construction of audiences which are affected by political interventions (Schneider and Ingram, 1993; Schneider and Sidney, 2009). Similarly, previous research suggests that the media’s construction of tobacco-related issues, including of policies aimed at tackling exposure to second-hand smoke (SHS), can impact public and political opinion. Assessing newspaper coverage of Dutch smoke-free legislation, for example, Nagelhout et al. (2012) suggest that media attention influenced SHS harm awareness and smokers’ support for legislation. Equally, Freeman et al. (2008) identify media coverage of the Australian smoking prohibition in vehicles as crucial in increasing public support for legislative measures.

It has been argued that investigating the social construction of target audiences can contribute to our understanding of policy debates (Schneider and Ingram, 1993). Previous social construction studies have primarily focused on policy document analysis (Chanley and Alozie, 2001; Donovan, 1993; Yoo, 2008), but scholars, including Pierce et al. (2014) and McAuliffe Straus (2004), also highlight media analysis as a valuable way of investigating the social constructions of target audiences. McAuliffe Strauss’ (2004) quantitative and qualitative study of the social construction of school desegregation in Los Angeles, for example, showed how newspaper media contributed to changes in audience understandings and beliefs of magnate schools policy over a period of three decades.

Target populations have been defined as ‘persons or groups whose behaviour and well-being are affected by public policy’, and social constructions as the cultural characterisations attributed to them (Schneider and Ingram, 1993). Schneider and Ingram’s (1993) theory of social constructions attempts to explain why some groups are presented as deserving or undeserving and how policy design can reinforce such perceptions. They assert that individuals tend to select information which re-affirms their existing attitudes and beliefs towards certain groups in society and that their view of others is thus highly subjective and context-specific (Munro and Ditto, 1997; Ritchie et al., 2010; Schneider and Ingram, 1993). Scholars also postulate that the public and political perceptions of target populations affect the behaviours and decisions of others, including political action which affects these population groups. Ingram et al. (2007) assert that ultimately, political decisions are dependent on whether the respective target populations are socially constructed and publicly perceived as powerful, vulnerable, deserving or undeserving.

Following such assumptions, Schneider and Ingram (1993) distinguish between four types of target populations: (1) advantaged groups who are powerful and positively constructed, (2) contenders who are powerful but negatively constructed, (3) dependents who are politically weak but positively constructed and (4) deviants who are weak and negatively constructed. They also postulate that those who are vulnerable, such as children, are likely to be seen as deserving of benefits and protection, whereas those viewed as deviants, such as criminals, are likely to be deemed unworthy and in need of punishment. Given that public officials are assumed to be strongly influenced by social constructions and the public representation of different population groups in their decisions to design, adopt and implement policies (Jørgensen, 2012), the media’s portrayal of target populations can be crucial in supporting the rationale for developing a specific policy (Schneider and Ingram, 1993) and have consequences for policy development and implementation (McAuliffe Straus, 2004).

Since 2006, when Scotland followed Ireland and became one of the first countries in Europe to implement comprehensive smoke-free legislation prohibiting smoking in enclosed public spaces, Scotland has been at the forefront of tobacco control policy. Scotland’s latest tobacco control strategy aims to reduce smoking prevalence to <5 per cent by 2034 and sets a target to reduce children’s exposure to SHS (Scottish Government, 2013). In tandem with this strategy, policies to reduce children’s exposure to SHS in vehicles have received increasing political attention (Scottish Government, 2013). A recent Action on Smoking and Health Scotland Survey found that 75 per cent of Scottish adults were in favour of legislation prohibiting smoking in vehicles carrying children under the age of 18 years (ASH Scotland, 2014), demonstrating high levels of support among the Scottish public for corresponding smoke-free laws. Building on the favourable public and political climate towards reducing children’s exposure to SHS in vehicles, Jim Hume, a Liberal Democrat Member of the Scottish Parliament (MSP), proposed a draft bill on 28 May 2013. The proposal was lodged on 30 January 2014 to the Scottish Parliament after achieving the necessary cross-party support from 44 MSPs (Scottish Government, 2014a), and the ‘Smoking Prohibition (Children in Motor Vehicles) (Scotland) Private Member’s Bill’ was introduced for consideration to the Scottish Parliament on 15 December 2014 (Scottish Government, 2014b). On 25 March 2015, the bill was formally backed by the Scottish Government. At present, the Scottish Parliament Health and Sports Committee is considering the evidence regarding the bill (Scottish Government, 2015), and it is intended that legislation will be implemented in 2016 (ASH Scotland, 2016). Both the UK government and the Welsh Assembly already implemented legislation to prohibit smoking in vehicles carrying children under the age of 18 years on 1 October 2015 (British Government, 2015).

Despite promising to increase understanding about the factors that might influence policymakers’ decisions on tobacco control issues, the social construction of target populations in debates on policies to reduce children’s exposure to SHS in vehicles has not been investigated. In an attempt to address this gap, this study investigates how children, smokers and smoking parents were presented in the media and how respective constructions may have been used to underpin arguments for or against policies prohibiting smoking in vehicles with a child present. The study, using data from the Scottish media as an example, thereby provides valuable global insights into on-going public debates on children’s exposure to SHS and the need to adopt policies to protect children from the harms caused by SHS. By reflecting on the opportunities and caveats of constructing target populations in the context of public health debates, this article helps to inform future advocacy on, and media coverage of, tobacco control and other public health issues.

## Methods

We carried out a media content analysis using a process of content analysis research outlined by Neuendorf (2002). The analysis of media coverage and textual data has been identified as a suitable method of measuring social constructions of target populations (McAuliffe Straus, 2004; Pierce et al., 2014; Schneider and Ingram, 1993). Building on two previous studies which provided an overview of arguments presented in UK newspaper debates on children’s exposure to SHS (Hilton et al., 2014a; Patterson et al., 2015), this study specifically examined Scottish newspaper articles to explore the representations of children, smokers and smoking parents and identify arguments used to support or oppose policies regulating smoking in vehicles. The Scottish case is particularly pertinent as Scotland was among the first European Union (EU) member states and the first country in the United Kingdom to publicly discuss legislation to prohibit smoking in vehicles carrying children (Scottish Government, 2013).

News articles published between 1 January 2004 and 16 February 2014 were identified in order to capture media debates prior to the 2006 enactment of Scottish smoke-free legislation in public places (Scottish Government, 2005) up until and briefly after Jim Hume’s 2014 ‘Smoking (Children in Vehicles) (Scotland) Bill’ was lodged in the Scottish Parliament. To cover a wide range of Scottish media debates and readership profiles, the three highest circulating Scottish national newspapers (National Readership Survey Online, 2015) and their corresponding Sunday editions, that is, the *Scotsman* and the *Scotland on Sunday*, the *Herald* and the *Sunday Herald*, and the *Daily Record* and the *Sunday Mail*, were selected. The sample consists of two ‘serious’ newspapers (the *Scotsman* and the *Herald*) which tend to be read by people from higher socioeconomic groups and one ‘tabloid’ newspaper (the *Daily Record*) with a readership profile with a higher proportion of those from lower socioeconomic groups (National Readership Survey Online, 2015). This typology has been used in other analyses of newspaper media discourse and represents a range of readership profiles diverse in terms of age, social class and political ideology (Hilton et al., 2014b).

Articles were identified using Nexis UK, adopting the search terms (where ‘!’ indicates a wildcard) ‘smok! OR tobacco OR cig! OR second hand smok! OR passive smok!’ AND ‘babies OR baby OR child! OR kid! OR infant! OR early years OR toddler! OR tot! OR parent! OR mum! OR dad! OR car! OR vehicle!’. Articles were excluded if (1) the content did not relate to children’s exposure to SHS, smokers exposing children to SHS or SHS in vehicles; (2) they were duplicate articles, letters, advice, TV guides, sport, weather, obituaries or review pages; (3) they related specifically to smoking during pregnancy; or (4) they were not published in the target newspapers. The filtering process left 131 Scottish news articles for detailed coding and analysis.

To develop an initial coding frame, 10 per cent of randomly selected articles were examined to identify key themes, which were tested on further batches of randomly selected articles. Key themes were contrasted and compared to identify patterns across the data and analyse the content of each theme in depth. The following three key themes emerged from the articles: (1) social construction of children, (2) social construction of smokers and smoking parents and the impact of their smoking on children and (3) arguments reporting on legislative and other actions to protect children from exposure to SHS in vehicles. Written summaries of the three thematic categories were developed independently by two researchers (J.B. and H.W.), any discrepancies were discussed and the coding frame was revised until agreement between researchers was reached. All articles were re-read and systematically coded according to agreed key themes.

## Results

Over the past decade, 131 Scottish news articles reported on children’s exposure to SHS. Of these, 49 articles were published in the *Scotsman*/*Scotland on Sunday*, 44 articles in the *Daily Record*/*Sunday Mail* and 38 articles in the *Herald*/*Sunday Herald*. The first news article which mentioned political intentions to prohibit smoking in vehicles was published in the *Scotsman* in 2008. Up until 2010, only three articles reported on the issue, suggesting scarce public attention. The policy debate appeared to gain momentum between 2010 and 2012 (n = 23), possibly in response to action by a number of non-governmental organisations, including ASH Scotland (2010) and the Royal College of Physicians (2010), calling on the Scottish and UK governments to outline their future strategies with regard to reducing SHS in vehicles. Articles during this period also covered a study, carried out in Scotland, by Semple et al. (2012), which demonstrated that ventilation systems and open windows were insufficient to protect children from exposure to SHS in vehicles. In 2013, all articles (n = 10) reporting on the smoking in vehicles debate were published in April and May, mirroring media attention around the draft proposal of the ‘Smoking (Children in Vehicles) (Scotland) Bill’. Five news articles were published in the first months of 2014, all of which focused on the final proposal of the bill.

News articles frequently depicted children as innocent victims of an unhealthy environment created by smoking adults. Articles mainly focused on the health risks to children from SHS exposure and the subsequent need to protect children as a group which was at particular risk of exposure. Of the articles which focused on the depiction of children and their exposure to SHS, most highlighted the health risks to children from exposure to SHS in the home or in vehicles, and fewer drew attention to the health risks from exposure to SHS in public places, including restaurants and playgrounds. In addition to focusing on children, articles frequently referred to smokers and smoking parents and the impact of their smoking on children. Here, several articles reported on smokers and smoking parents in a factual manner, that is, without evoking feelings of vulnerability or sympathy, but smokers and/or smoking parents were also frequently presented in a negative light. In rare cases, articles highlighted the need to help smokers to quit. Finally, almost one-third of all newspapers covered legislative and other actions to protect children from exposure to SHS in vehicles. Table 1 provides a summary of themes identified in the articles and the frequency in their reporting. The following sections elaborate on each of the identified themes in more detail and provide quotes to illustrate the themes.

**Table 1.** Themes identified in all articles (n = 131).

Themes and subthemes	Number of articles
Representations of children as innocent victims of an unhealthy environment created by smoking adults	111
Factual presentation of health risks of children’s exposure to SHS	91
Portrayal of children as helpless	106
Portrayal of children as active agents	5
Representations of smoking adults and the impact of their smoking on children	100
Factual presentation of smokers and smoking parents	99
Negative portrayal of smokers and smoking parents	29
Need to help smokers quit	15
Portrayal of smokers as protecting children	2
Presentation of legislative and other actions to protect children from exposure to SHS in vehicles	62
Legislative action	42
Other action	35

SHS: second-hand smoke.

Numbers of all articles, themes and subthemes do not add up as often several themes and subthemes were covered in one article.

### Representations of children as innocent victims of an unhealthy environment created by smoking adults

The first key theme to emerge from the articles focused on how children were socially constructed as a target population. A majority of articles drew attention to children as a particularly vulnerable group, stressing the comparatively stronger negative health effects of SHS for children. It was emphasised that children were especially affected by exposure to SHS, with advocates arguing that children ‘are the most vulnerable’ (Journalist, *Daily Record*: 28 January 2005), ‘at a particular risk’ (Elspeth Lee, Cancer Research UK, *Sunday Herald*: 3 February 2008) and, due to exposure and vulnerability, ‘more likely than adults to develop health conditions and illnesses’ (Dr Sean Semple, University of Aberdeen, *The Scotsman*: 16 October 2012). Frequently, long-term health effects were highlighted and respective claims underlined by referencing health professionals and scientific evidence. One doctor, for instance, was quoted in the *Daily Record* claiming that ‘children who suffer from passive smoking are more likely to develop a range of conditions, including asthma, glue ear and meningococcal meningitis’ (Dr Craig Lennox, *Daily Record*: 29 June 2010).

In addition to providing factual evidence of children’s comparatively higher vulnerability to SHS, articles were found to employ strong emotional language when describing children’s experiences of exposure to SHS. Journalists from different newspapers portrayed being exposed to SHS as a ‘distressing’ (Liam McDougall, Health Correspondent, *Sunday Herald*: 5 September 2004) and ‘shocking’ (Journalist, *Daily Record*: 29 June 2010) experience, with tobacco smoke being ‘subjected’ (Andrew Denholm, Political Correspondent, *The Scotsman*: 8 April 2004) upon children, who were ‘forced to endure polluted car rides’ (Dr James Cant, head of British Lung Foundation Scotland, *The Scotsman*: 7 September 2011) or ‘forced to become passive smokers’ (Journalist, *The Scotsman*: 5 January 2005). News articles claimed that ‘kids suffer more from passive smoking’ (Journalist, *Daily Record*: 24 March 2010) and ‘do not have the choice of breathing in fumes’ (Journalist, *The Herald*: 1 July 2005). These claims were especially promoted in relation to exposure to SHS in vehicles, where it was argued that ‘children were vulnerable in cars [as they] are unable to remove themselves’ (Alex Cunningham, Labour MP, *Daily Record*: 23 June 2011) effectively being ‘trapped in cars filled with smoke’ (Jim Hume, Liberal Democrat MSP, *Daily Record*: 29 April 2013).

The majority of articles portrayed children as unable to be their own advocates. Speaking at a British Medical Association conference, Dr Douglas Noble, a member of the public health committee, was quoted in the *Scotsman*, for example, as arguing that ‘86 per cent of children wanted people to stop smoking in vehicles [… but] heartbreakingly, were too afraid to ask adults to stop smoking’ (*The Scotsman*: 30 June 2011). Only a very small number of articles (n = 5) mentioned children’s ability to voice concerns about their own exposure to SHS and their parents’ smoking, highlighting their attempts to ‘nag’ and ‘lecture’ adults and other children about the harms of smoking. An article in the *Herald*, for example, cited a 13-year-old boy who was not only convinced that he would never smoke but also educated his peers on smoking-related issues: ‘I show the primary pupils all the chemicals in tobacco which are dangerous’ (David Low, *The Herald*: 6 June 2007). Similarly, a 10-year-old girl was reported in the *Daily Record* as raising the issue of smoking with one of her parents and challenging them about their health behaviour by saying, ‘Smoking can kill do you know? Why do you do it?’ (Courtney Hewitt, *Daily Record*: 10 January 2012).

### Representations of smoking adults and the impact of their smoking on children

A second key theme evident throughout the articles focused on the social construction of smokers and smoking parents as target populations of smoke-free legislation. Many articles reported about the risks deriving from smokers and smoking parents on children’s health in a factual manner and without including judgement or value statements. For example, a *Daily Record* journalist wrote, ‘Most new parents know it’s not advisable to smoke in front of their babies, but passive smoking can be harmful to children of any age’ (Journalist, *Daily Record*: 18 June 2004), while a journalist in the *Herald* commented, ‘Living with a smoker […] worsens the severity of asthma attacks and can lead to ear infections [in children]’ (Journalist, *The Herald*: 19 July 2011). Almost a quarter of all newspaper articles (n = 29), however, portrayed smokers as egoistic, irresponsible and willingly inflicting harm on vulnerable children, complementing the social construction of children as powerless, passive sufferers who were in need of compassion and care. Of these articles, many used very strong wording to draw attention to smokers’ and smoking parents’ alleged lack of concern. Articles typically constructed these individuals as ‘failing to protect their children from the harmful effects of nicotine’ (Tara Womersley, Health Correspondent, *The Scotsman*: 11 February 2004), ‘stubborn’ (Journalist, The *Scotsman*: 1 March 2006) and ‘reluctant to stop […] despite it being clear that their habit is affecting not only their own health but also that of their children’ (Dr Dean Marshall, *Daily Record*: 22 May 2004). Some journalists described smokers as ‘persistent offenders’ (Journalist, *The Scotsman*: 1 March 2006), who were ‘refusing’ to stop smoking (Journalist, *Daily Record*: 29 June 2010) and ‘slowly ruining the health of those they love most’ (Journalist, *Daily Record*: 20 January 2011). Articles construed smoking parents as needing to ‘take more responsibility’ (Dr Steve Field, *Daily Record*: 9 August 2010), with stronger worded articles arguing that smoking parents ‘should be ashamed’ (Valerie Simpson, Children with Cancer and Leukaemia Advice and Support for Parents, *Sunday Mail*: 23 September 2012) or portraying them as ‘unrepentant’ (Journalist, *Daily Record*: 29 June 2010). Individuals who smoked in vehicles carrying children were presented as ‘forcing [children] to breathe in dangerous levels of poison’ (Journalist, *Daily Record*: 20 January 2011). Journalists from different newspapers applied even more drastic language, portraying smoking parents as committing a form of ‘child abuse’ (Journalist, *Daily Record*: 9 August 2010), stating that ‘millions of youngsters are being put at risk by their nicotine-addict parents’ and calling for ‘a crackdown on smokers, including a ban on mums and dads’ (Journalist, *Daily Record*: 24 March 2010), who ‘don’t value their children’s health’ (Muriel Gray, Journalist, *Sunday Herald*: 21 June 2006).

Only a small number of articles acknowledged that parents were in need of ‘encouragement’ (Journalist, *Daily Record*: 29 June 2010) and ‘support to help stop smoking’ (Journalist, *The Herald*: 5 June 2008). Such articles focused on health education as an approach to supporting smokers and smoking parents in quitting. One article in the *Daily Record*, for example, quoted Dr Laurence Gruer, Director of Public Health Science National Health Service (NHS), as stating that ‘we should be trying to get through to parents at every opportunity just how unhealthy smoke is to their children’ (*Daily Record*: 8 January 2006). Another article in the *Scotsman* cited Anna Johansson, a public health specialist at Linköping University, Sweden, as saying that many ‘parents are not as careful as they think’ when taking general measures to reduce their children’s exposure to SHS (*The Scotsman*: 11 February 2004). Comments which suggested that telling smokers and smoking parents to stop smoking was sufficient to counter the problem uncovered a failure to acknowledge the addictive nature and complexity of smoking.

Contrasting the generally negative portrayal of smokers, two articles highlighted that smoking parents did care for their children and tried to be responsible. These articles drew attention to ex-smokers who had given up smoking for the ‘sake of their kid’s health’ (Journalist, *Daily Record*: 10 January 2012). Journalists reported that parents were concerned about the detrimental impact of their smoking, both on their own and their children’s health, and that corresponding concerns had resulted in action to protect children from exposure to SHS, particularly in the home environment. For example, one article in the *Scotsman* quoted a 23-year-old mother as saying, ‘When you see that the smoke is hurting your child […] there is no excuse – you have to do something about it’ (Kelly Rickman, *The Scotsman*: 19 May 2005). The other article presented a 40-year-old father as having been persuaded to quit smoking by concerns of not being able to see his children grow up due to the likelihood of early mortality: ‘My wife had pointed out if I carried on smoking I wouldn’t get to see the kids grow up … Now I’m looking forward to a healthier future with my family’ (Steve Watson, *Daily Record*: 10 January 2012).

### Arguments reporting on legislative and other actions to protect children from exposure to SHS in vehicles

The third theme to emerge from the articles centred on various arguments used in support of, or opposition to, smoke-free legislation in vehicles carrying children under the age of 18 years. This last theme was identified as particularly important to the study’s aim of exploring the social construction of target populations in the debates on regulating smoking in vehicles because of its contribution to understanding whether specific populations were constructed as deserving or undeserving of corresponding policies. Over the 10-year period, legislative or other actions were mentioned in 62 articles. A variety of potential solutions to protect children from SHS were discussed throughout the newspaper articles. Almost one-third of all articles (n = 42) centred on legislative action to prohibit smoking in vehicles, whereas 35 articles concentrated on other potential action, including education and advertising campaigns, and cessation programmes. Articles related to legislation more commonly included arguments that were supportive (n = 35) than critical (n = 22) of legislative proposals. Furthermore, it is of note that, although there tended to be multiple perspectives highlighted within a single article, supportive arguments were given more weight and space throughout the articles than arguments critical of legislation, with critical perspectives often only highlighted as one or two sentences at the end of an article.

Supporters of legislation to prohibit smoking in vehicles included politicians, government officials, representatives of public health organisations and charities, doctors and scientists. Arguments in favour of prohibiting smoking in vehicles covered the need to protect children’s health from the negative effects of SHS (an argument raised in 33 articles), the importance of legislation in changing attitudes towards smoking and the dangers of SHS (n = 9) and the ease with which legislation could be enforced (n = 4). Supporters who utilised a ‘health’ frame depicted legislation as part of a comprehensive tobacco control strategy and emphasised the harms of SHS exposure to children and the likely health benefits of legislation. Seemingly aware of the persuasiveness of such a frame, Jim Hume was cited in the *Herald* as stating that the issue ‘should be treated as a health issue’ (*The Herald*: 31 January 2014). As illustrated by the following quote by British Lung Foundation Scotland’s head James Cant, legislative action was also frequently promoted as a way to change attitudes to smoking: ‘This is […] an attempt to change [smokers’] smoking habits and, again, the evidence suggests legislation is the most effective method of bringing about this kind of behaviour change’ (*The Scotsman*: 29 May 2013). Several news articles drew attention to the need to protect children from harms caused by SHS. Following such discourse, public health advocates stated that ‘Scottish children deserve protection from the invisible dangers of second-hand smoke’ (Dr James Cant, *The Herald*: 11 February 2014) and that as ‘you can’t inflict smoke on your colleagues at work anymore […] why should we treat our children’s health as a lower priority?’ (Prof Terrence Stephenson, head of the Royal College of Paediatrics and Child Health, *Daily Record*: 18 June 2009). Supporters depicted legislative measures to prohibit smoking in vehicles with a child present as ‘safeguarding the rights of children’ (Jim Hume, Liberal Democrat MSP, *The Scotsman*: 29 May 2013) and ‘help[ing] prevent hundreds of thousands of children from being exposed to second-hand smoke in the car […which] is a crucial child protection measure’ (Dr Penny Woods, chief executive of the British Lung Foundation, *The Scotsman*: 11 February 2014). Measures were also supported for de-normalising smoking by ‘mak[ing] it clear to the transgressors that they are doing something which society at large disapproves of’ (Muriel Gray, Journalist: *The Sunday Herald*: 21 June 2009) and getting the messages across that ‘a private vehicle is one of the few places a child can still be legally exposed to tobacco smoke’ (Jim Hume, Liberal Democrat MSP, *The Herald*: 28 May 2013). Supporters of legislation further argued, using other legislatures such as Australia, South Africa and Canada as examples, that ‘research in other countries shows that this [law] can be enforced successfully’ (Jim Hume, Liberal Democrat MSP, *The Herald*: 28 May 2013).

Those who opposed legislation largely reiterated previously employed arguments to counter tobacco control measures and seemingly intended to cast scepticism on the likely benefits of legislative measures and highlight the unintended consequences a ban would have on public revenue (cf. Savell et al., 2014). The tobacco industry funded lobby group FOREST was by far the most cited proponents of such arguments, being mentioned in 17 of the 22 news articles that raised arguments which were critical of legislation. In fact, few other critics were cited. Criticism of intentions to prohibit smoking in vehicles carrying children included that such policies constituted intrusions into individual freedom and the private space (an argument raised in 12 articles), were unenforceable (n = 10), the wrong method when aiming to change attitudes (n = 4), not based on evidence (n = 3) and not supported by the public (n = 2). Simon Clark, FOREST’s main spokesperson, was cited in the *Herald*, for example, as claiming that research evidence ‘doesn’t support the argument smoking in cars is a serious health risk to children’ (*The Herald*: 16 November 2011) and in the *Scotsman* as arguing that evidence use by supporters of legislative action ‘borders on scaremongering’ (*The Scotsman*: 20 January 2011). Critics claimed that legislation ‘would be very difficult to enforce’ (Simon Clark, *The Scotsman*: 29 May 2013) and constituted an ‘intrusion’ of smoker’s civil liberties (Deputy Prime Minister Nick Clegg, Liberal Democrats, *The Scotsman*: 11 February 2014). Following the latter argument, a *Scotsman* journalist commented, ‘We have the right to make choices free of state interference. Banning smoking in cars is an authoritarian step too far’ (*The Scotsman*: 16 October 2012). Furthermore, opponents often suggested advertising campaigns and education programmes as alternative, non-legislative means to inform smokers about the dangers of SHS and encourage them to cut down smoking in certain contexts, claiming that such measures were more appropriate than legislation.

## Discussion

This article provides some of the first insights into the social construction of children, smokers and smoking parents in the Scottish media in the decade leading up to the 2014 Scottish Parliament draft Member’s Bill proposal to prohibit smoking in vehicles carrying children. Following Yoo’s (2008) assertion that individuals cited as experts in newspaper articles are instrumental in the social construction of deserving and undeserving social groups, our analysis shows that journalists, public health officials, advocates and politicians were the main participants in media debates and considerably contributed to social constructions of children, smokers and smoking parents. Supporters of legislation to restrict smoking in vehicles employed a health frame to strengthen their arguments and promote legislative action as a reasonable and justifiable solution. Opponents of legislation, in contrast, avoided health-related debates, instead employing libertarian and ‘regulatory redundancy’ frames, arguments that corresponding laws would be difficult to enforce and constitute an indefensible intrusion into the private space to counter-regulatory measures and other arguments that had previously been used by those resisting tobacco control measures (cf. Savell et al., 2014; Weishaar et al., 2012). Our study also highlights opponents’ difficulties in arguing against the health frame and legislation to protect children from SHS, thereby supporting a previous analysis of Australian news media debates, which suggests that portraying children as a vulnerable group creates ‘an almost invincible powerful sub-text’ to legislation on smoking in vehicles which critics are unlikely to oppose (Freeman et al., 2008).

Our study further demonstrates that media content analysis can increase understanding of the social construction of societal groups as deserving and undeserving of public health policies and the rationales that underpin these constructions. Scholars have argued that the way in which target populations are constructed impacts public opinion and public officials’ aptitude or reluctance to advance and adopt policies (Schneider and Ingram, 1993). Schneider and Ingram (1997: 124) argue that ‘much of the dynamics of policy design for dependent people hinges on separating the deserving from the undeserving’ and that groups which are presented as powerless and deserving of care are likely to be the beneficiaries of protective, albeit sometimes patronising policies. Our analysis of Scottish media articles on policies restricting smoking in vehicles carrying children shows that children were constructed as vulnerable and deserving of protection and smokers and smoking parents as a population whose harmful behaviour was in need of regulation. The clear attribution of burdens and benefits to these two target groups mirrors Schneider and Ingram’s typology.

Our findings further support work by Malone et al. (2000) who argue that journalists cite individuals who are perceived as experts to legitimise the ‘facts’ used in news stories on public health issues. Malone et al. (2000) also argue that those ‘facts’ remain open to further social construction. Our study is in line with such research and highlights that along with the media and journalists, scholars working on public health issues contribute to constructing knowledge of target groups affected by public policies, with constructions being constrained by the contexts in which those who contribute to such constructions operate. This means that the social construction of public health issues is influenced by a variety of issues, with the interpretations and actions by journalists, public health experts and others, while playing important roles in influencing public health debates and public and political attitudes and behaviours (Freeman et al., 2008; McAuliffe Straus, 2004; Malone et al., 2000) constitutes only one of a wide range of factors.

Our analysis on the social construction of target audiences raises further issues which should be considered by those involved in debates on public health policy. First, in the majority of articles, advocates spoke on behalf of children, whereas only 5 of 131 articles gave children an active voice. In contrast to the prominent discourse, the few articles which quoted children directly tended to portray children as empowered and as able to raise concerns with their parents and other adults about their smoking. These articles reflect, to some extent, previous work which argues that children are significantly aware and concerned about the harms of smoking and can add considerable value to policy processes to regulate exposure to SHS (Porcellato et al., 2002; Malone et al., 2002; Rouch et al., 2010; Woods et al., 2005).

Complementary to such findings, Badham (2004: 147) specifically recognises the need to include children and young people in decision-making processes on issues that affect them, suggesting that policies aimed at protecting children may otherwise become ‘vulnerable to tokenism and adult manipulation’. Based on such research and our findings, it can be assumed that giving children an opportunity to express their awareness of, and concerns about, the harms caused by smoking and exposure to SHS in policy and media debates could emphasise the need for action while benefiting and empowering those who are most affected by harmful actions. The added value of involving young people in the development of policies, services and public communication with regard to tobacco control has been acknowledged by the Scottish Government, resulting in the establishment of the Youth Commission on Smoking Prevention (Scottish Government, 2013). Further action in this area would be welcomed.

Second, previous research suggests that smoke-free legislation has led to lower acceptability of smoking and the marginalisation and stigmatisation of smokers (Ritchie et al., 2010; Rooke et al., 2013). The identification of only two articles in this study which quoted smokers directly is consistent with Malone et al.’s (2000) US newspaper analysis of SHS debates which found that smokers tended to be spoken about, rather than be actively involved, in media discussions, subsequently underpinning their increased marginalisation in societal debates. While focusing on children’s vulnerability and stigmatising smokers might be potent tools to de-normalise smoking and promote stricter tobacco control policies (Bayer, 2008; Malone et al., 2000; Ritchie et al., 2010; Rowa-Dewar and Ritchie, 2014; Stuber et al., 2008), such social constructions have ethical implications. Bayer (2008) argues that there may be circumstances when stigmatisation is morally defensible, but highlights that corresponding decisions should be carefully considered, especially given the over-proportionate burdens of tobacco control measures on those at the bottom of the social ladder. Public health advocates have a responsibility to contemplate the effects of stigmatisation on behaviour, the likelihood of this approach for entrenching smokers’ behaviours and the extent of the suffering that stigmatised individuals will have to bear (Bayer, 2008).

Future media coverage of tobacco regulation will provide opportunities to give more active voices to, and empower, both those who are affected by harmful behaviour and those who are the primary targets of political action. Media debates could also help to draw attention to the need to address the underlying social determinants of smoking, such as social inequalities in health, when developing solutions to address poor health (Giesinger et al., 2013; Scottish Government, 2008). We hope that a better understanding of the advantages and caveats of social constructions of target audiences will help to make informed, strategic decisions and cover tobacco-related issues in ways which advance public health while avoiding unintended stigmatisation and promoting inclusive debates.

While our analysis does not allow us to draw conclusions about the impact of the social construction of children, smokers and smoking parents on understanding of, and support for, and the development of legislation or on the media’s direct influence on increasing the pertinence of the issue in the public and political sphere, it is reasonable to assume that the way in which target populations were socially constructed in the media might have attracted attention to children’s exposure to SHS in the vehicle and contributed to perceptions of children as powerless and deserving of political action, thereby helping to build a favourable public climate for legislative action as a potential solution and increase political will to move a proposal to regulate smoking in vehicles up the political agenda. By presenting data from the first study on the social construction of target populations in public debates leading up to the 2014 ‘Smoking Prohibition (Children in Motor Vehicles) (Scotland) Bill’, this study provides a valuable addition to the public health literature and on-going global debates on the regulation of smoking in vehicles carrying children. The findings are particularly pertinent as, apart from Cyprus, Italy and some parts of the United Kingdom, no EU member state has currently implemented legislation to protect children from SHS in vehicles (Bennett, 2015; Campbell, 2014). Following the UK example, the German Commissionary for Drugs (Spiegel Online, 2015) and the German network for tobacco control, Aktionsbündnis Smoking eV (ABNR, 2015), have called for a ban on smoking in vehicles when children are present. Elsewhere in Europe, the Belgian Government has been reported to be considering a move to ban smoking in vehicles carrying minors (McNally, 2015). Across the rest of the world, the ban exists in parts of Canada, the United States and Australia, while South Africa and Bahrain have prohibited smoking in vehicles when children are present, and Mauritius has enforced an outright ban on smoking in vehicles. Considering these developments, the debates presented here are likely to have wider implications and relevance for emerging national and international public and political tobacco control debates.
